# Can colorectal cancer survivors recall their medications and doctor visits reliably?

**DOI:** 10.1186/1472-6963-12-440

**Published:** 2012-12-02

**Authors:** Louisa G Gordon, Tania Patrao, Anna L Hawkes

**Affiliations:** 1 Griffith University, Centre for Applied Health Economics, Griffith Health Institute, University Drive, Meadowbrook, Brisbane, Queensland, 4131, Australia; 2 Queensland Institute of Medical Research, Genetics and Population Health Division, PO Royal Brisbane Hospital, Herston, Brisbane, Queensland, 4029, Australia; 3 Viertel Centre for Research in Cancer Control, Cancer Council Queensland, Gregory Tce, Spring Hill, Brisbane, Queensland, 4006, Australia; 4 School of Public Health, Queensland University of Technology, Kelvin Grove, Brisbane, Queensland, 4059, Australia

## Abstract

**Background:**

The evidence on the agreement between self-reported health resource use and administrative records is mixed and no gold standard exists. The objective of this study was to assess self-reported general practitioner (GP) and specialist doctor visits, as well as medication use via telephone interview against national insurance administrative data for colorectal cancer survivors.

**Methods:**

In a sample of 76 adults recently diagnosed with colorectal cancer, data was abstracted from telephone survey items on GP visits, specialist visits and medication use over the previous six months and compared with data on the same individuals from administrative data. Intraclass correlation coefficients (ICC) were used to assess the reliability of frequency of visits and kappa statistics were derived for four broad categories of medicines used for gastrointestinal conditions, cardiovascular disease, psychological conditions and chronic obstructive pulmonary disease. Logistic regression was undertaken to assess factors associated with agreement (yes/no) between the two data sources for doctors’ visits.

**Results:**

Good agreement was found for GP visits (ICC 0.62, 95%CI: 0.38, 0.86) and specialist visits (ICC 0.73, 95%CI: 0.56, 0.91) across the two data sources. When costs were assigned to frequencies, mean costs for the two methods were not significantly different over six months. Over-reporting was more common among men and participants with frequent doctor encounters. Large discrepancies between self-reports and administration records were found for broad types of medications used (44% agreement, kappa 0.13).

**Conclusion:**

Self-reported frequency of doctor’s visits using telephone interviews may be a reasonable substitute for administratively recorded data however, medication use by self-report appears to be unreliable. Administrative records are preferable to self-report for health service use in colorectal cancer survivors with high and complex service needs.

## Background

During the economic evaluation of health services, health care resources are required to be quantified and valued in monetary terms. The accurate estimation of these costs is crucial within economic analyses, however there are no standardized methods for collecting the quantities of health service use at the patient level. This is mostly because every study setting is unique, and the collection of this data is often constrained by the study design as well as participant burden considerations [1,2]. Health resource use may be collected through a variety of means including patient chart reviews, administrative records at hospitals, clinics or insurance companies or by participant-reported methods, (self-reported using surveys, interviews or diaries). By necessity, the linkage of various sources is often required to capture the full extent of resources used [3].

Each method comes with its own set of advantages and limitations. Self-reported methods are open to poor recall or memory decay, interpretation difficulties, and increased participant burden. However, these methods are often cheaper, more efficient, and useful when multiple health services and locations are used for a particular health condition. Medical chart review is a relatively labour-intensive method. There may be difficulty in accessing medical charts and special expertise may be required for interpreting the data, however a wide range of clinical data is available. Administrative records are not designed for research projects and therefore not all relevant data is likely to be available and there is the problem of timely access, cost, legal and privacy limitations and an unknown degree of programming or coding errors. There is currently no gold standard or benchmark although many researchers suggest administrative records are more likely to be accurate for encounters with health services [2,3]. Automated administrative data sets also allow large numbers of individuals to be analysed and compared with other data sources. Moreover, the availability of data linkage systems and electronic health records will facilitate large-scale, population-based research and health service evaluation, subject to the issues of missing data, confidentiality and errors [4].

Many studies have investigated the extent of agreement between at least two of the available methods in different populations, with or without certain health conditions, to decide on their exchangeability. The discordance between different methods has been attributed to the types of services studied, the recall period, socio-demographic profile of the target population, poor health status and symptoms, high stress or high treatment needs, errors in administrative records, incorrect recording of records and patients misspecification of a treatment or service [2,5]. Importantly, the reliability and validation of readily available instruments for researching self-reported health care utilisation is an area still developing with standardized methods gradually improving, see http://www.dirum.org. The task is particularly challenging when there is a high volume of resource types and quantities and considering adequate coverage of resource types across many different diseases and patient groups.

In general, studies have shown poorer agreement for participant-supplied data against routinely collected administrative data sources among elderly participants (due to high service needs and potential memory decline), males, those experiencing high frequency of visits, health service contacts of a non-serious nature, poor health status and collecting data over longer recall periods (e.g., 12 months) [2,5]. Several reports have also shown good reliability of self-reported data in relation to administrative data [6-9] and particularly for hospitalisations [2,6,8]. It has been suggested that since generalisation across studies is problematic due to differences in populations, diseases and recollection periods, health service researchers should address the internal validity of their own health service use data, uncover any systematic bias, its potential uncertainty and characterise the ensuing implications [5].

To this end, in the context of a larger cost-utility analysis for assessing the costs and benefits of a lifestyle intervention for colorectal cancer survivors (CanChange) [10], we tested the consistency and validity of self-reported GP and specialist visits over six months, and medication use against linked administrative data from the national Medicare Benefits Scheme among a sub-sample of participants (n = 76). In addition, we assessed factors that potentially influenced disagreement in responses across the two methods.

## Methods

### Sample and data collection

The study was approved by the Human Research Ethics Committees of the University of Queensland's Behavioural and Social Sciences Ethical Review Committee (approval number 2007000656), Medicare Australia and 18 private and public hospitals throughout Queensland. A sub-sample of 76 consecutive participants (approximately 20%), ensuring equal numbers of males and females, were selected from the CanChange study, a two-armed prospective randomised controlled trial enrolling 410 adults newly diagnosed with colorectal cancer to the intervention or control group. The trial aims to assess an intervention designed to promote healthy lifestyle changes to participants by trained and qualified health professionals (‘Health Coaches’) via the telephone. Full details of the CanChange study are previously reported [10]. Health resource data was obtained as part of a cost-utility analysis using trial data from the CanChange study. Participants completed assessments at baseline (Time 1), post-intervention or 6 months follow-up (Time 2, the primary endpoint), and at 12 months follow-up to assess longer term effects (Time 3). The present study uses data collected over a six-month period from baseline to six months follow-up. The data relates to the total study time period from March 2009 to November 2010.

Administrative data was retrieved from Medicare Australia, Australia’s national insurance system and provider of subsidised health professional consultations, medical procedures and medications to all Australian citizens. Medical consultations and procedures are listed on the Medicare Benefits Schedule (MBS) and medications are listed on the Pharmaceutical Benefits Scheme (PBS). Study participants were required to provide written informed consent for Medicare to release their records to the researchers, separately to the CanChange study consent form. The cost to researchers to obtain the MBS and PBS data was AU$3,450 for the sub-sample. For medication data, due to the very high volume of medicines used by the participants, we focussed on four broad medication groups that in turn, were guided by their therapeutic classes of action. The four groups included medicines for gastrointestinal problems, cardiovascular disease, psychological conditions and chronic obstructive pulmonary disease. These groups were specifically chosen as being the most amenable to change as a consequence of the CanChange lifestyle intervention components; healthy eating, increased exercise, gastrointestinal symptoms, stronger psychological well-being and smoking cessation. The agreement between the two methods across the four groups was assessed.

Data on self-reported health care resource use was collected by qualified researchers during telephone interviews. The questionnaire was developed and pilot tested with the assistance of a convenience sample of cancer survivors. Questions related to health service utilisation and medication use were posted ahead of the telephone interview time to encourage participants to prepare their answers, by gathering necessary supporting records or receipts of consultations and medication prescriptions. If respondents were unsure of any questions, clarification was provided during the interview. The following question was asked relating to medications during the past six months: *Are you currently taking any medications that have been prescribed for you by a doctor or specialist?* If the respondent answered affirmatively, further details were asked to be completed in a table format for medication name and dose per day. For doctor visits, the following question was asked; *Have you visited a doctor (general practitioner or specialist) in the last 6 months?* If the respondent answered affirmatively, details were asked in a table format for doctor’s name, doctor type, suburb of practice, number of times visited. Similar questions were asked for visits to other health professionals, hospital inpatient and outpatient stays and community health services. A copy of the questions relating the health care use in this study is available from the authors upon request (Additional file 1).

### Analyses

Descriptive statistics (n,%) were calculated for the two data sources for *any* doctor visits or medications (yes/no) and *frequency* of doctor visits. In addition, due to the vast number of different medications possible in our sample, we categorised the survey and MBS medication data into categories according to broad therapeutic class (e.g., analgesics, antihypertensives, acid reflux). The level of agreement for categorical data using the proportion of absolute agreement and beyond chance agreement was according to Cohen’s kappa statistic (κ). For continuous data on frequencies of doctor visits, the random effects intraclass correlation coefficient (ICC) was used. The following guide was used for interpreting the strength of agreement using both the κ and ICC : <0.2 = poor, 0.21-0.40 = fair, 0.41-0.60 = moderate, 0.61-0.80 = substantial, and >0.81 = almost perfect [5].

Scatter plots were used to assess the spread of responses for frequency data. To investigate which factors might be associated with absolute agreement, under- or over-reporting, using the administrative MBS and PBS data as the reference source, chi-square tests were performed for socio-demographic and disease-related variables. Due to the small sample, collapsing some variable categories into dichotomous responses occurred. New variables were also constructed for frequency (high/low) for doctor visits (separately for GPs and specialists) based on the median number of visits. Following these bivariate analyses, a dichotomous variable of agreement (yes/no) was constructed and logistic regressions were performed using all statistically significant variables detected from the bivariate analyses. Finally, we assessed differences when costs were applied to the doctor visits for the survey methods against the MBS benefits paid. We applied an average GP unit cost across MBS A1 attendance categories ($39) and average specialist unit cost across A2-A4 categories ($55). As costs per participant were skewed, bootstrapping using 1000 replications and the bias-corrected method was employed to assess differences in costs across the two data collection methods [11].

## Results

In our sample of 76 participants, the mean (SD) age was 65 (9.8) years and 32% were aged between 71-80 years (Table 1). Men and women were represented equally, 68% lived with a partner, 70% had private health insurance, 47% had higher education qualifications and 49% were retired from the work force. Twenty-four percent had Duke’s stage C cancer (severe disease), 54% had received open surgery versus 43% for laparoscopic surgery. Participants were also commonly treated with adjuvant chemotherapy (59%) and/or radiotherapy (84%) (Table 1).

**Table 1:** Profile of participant characteristics (n = 76)

**Socio-demographics**	**n**	**%**	**Disease profile**	**n**	**%**
Sex			Tumour site		
Male	38	50%	Rectum	17	22%
Female	38	50%	Colon	59	78%
Age (mean 65 years, SD 9.8 years)			Dukes Stage		
31-40	2	3%	A	17	22%
41-50	4	5%	B	22	29%
51-60	14	18%	C	18	24%
61-70	32	42%	Unknown	19	25%
71-80	24	32%	Type of surgery		
Country of birth			No surgery	4	1%
Australia	59	78%	Open surgery	41	54%
UK	7	9%	Laparoscopic	33	43%
New Zealand	5	7%	Polypectomy only	1	1%
Other	5	7%	Chemotherapy		
Living arrangements			Yes	45	59%
Single live alone	15	20%	No	31	41%
Single live with friends/family	5	7%	Radiotherapy		
Single parent	2	3%	Yes	64	84%
Couple not living with children	38	50%	No	12	16%
Couple Living with Children	14	18%	Stoma		
Other	2	3%	No	62	82%
Private health insurance			Temporary	9	12%
No	16	21%	Permanent	5	7%
Yes	60	79%	Comorbid conditions		
Highest education			Diabetes	9	12%
Primary School	5	7%	High cholesterol	26	34%
Secondary School	35	46%	Hypertension	29	38%
Higher Education	36	47%	Angina	1	1.3%
Employment situation			Peripheral vascular disease	6	8%
In paid work	18	24%	Lung disease	11	14%
Work without pay	4	5%	Depression, anxiety, nervous	11	14%
Unemployed	2	3%	Gastrointestinal ulcer	4	5%
Retired	37	49%	Arthritis	26	34%
Permanently unable to work	2	3%	Osteoporosis	8	11%
Other	11	14%	Kidney disease	3	4%
Missing	2	3%	Any other cancer	21	28%
Total household income					
<$25,000	19	25%			
$25,001 - $40,000	21	28%			
$40,001 - $65,000	10	13%			
$65,001 - $100,000	9	12%			
>$100,000	6	8%			
not stated	11	14%			

### GP visits

**Figure 1:**
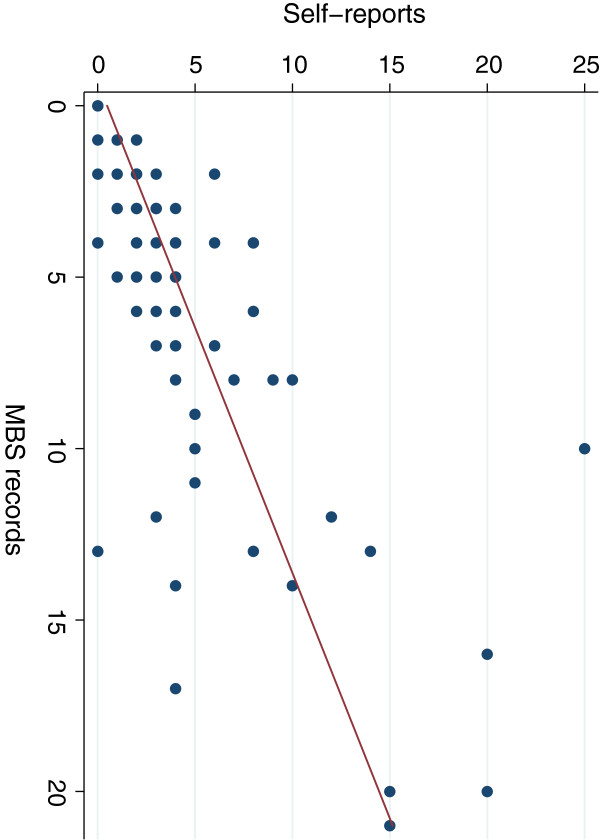
Scatterplot of frequency of self-reported GP visits from telephone interview and MBS GP visit records (n = 76).

The distributions of the frequency of GP visits were right-skewed and showed a median of 3.0 visits for self-report data versus 4.5 for MBS records (Table 2). Overall, the mean difference was 1.34 fewer visits for survey reports than MBS records, indicating participants’ under-reported visits by 1 to 2 visits, on average over six months. Perfect agreement was found in 20 (26%) participants, over-reporting in 14 (18%) participants and under-reporting in 42 (55%) participants (Table 2). When a margin of ±1 visit was allowed, 53% reported perfect agreement with MBS records and 64% within ±2 visits (Table 2). The ICC was 0.62 (95%CI: 0.38, 0.85) indicating substantial agreement but ranging from poor to substantial. Figure 1 shows better agreement across the two methods with fewer visits to recall over the past six months and excess self-reported visits with more frequent visits. Bivariate analyses identified that males and individuals not in paid work were significantly more likely to under-report visits and more over-reporting occurred with higher frequency of visits (> 3 visits) (Table 3). These factors remained statistically significant in a multivariate logistic regression model (Table 4).

**Table 2:** Agreement between self-reported and administrative reports on the frequency of GP and specialist visits (n = 76)

	**GP visits**	**Specialist visits**
Perfect agreement	20 (26%)	12 (16%)
Over-reporting	14 (18%)	34 (45%)
Under-reporting	42 (55%)	30 (39%)
Agreement within ± 1 visit margin	40 (53%)	27 (36%)
Agreement within ± 2 visit margin	49 (64%)	39 (51%)
Intraclass correlation coefficient (95% CI)	0.62 (0.38, 0.86)	0.73 (0.56, 0.91)

**Table 3:** Associations between socio-demographic and clinical factors and disagreement category by health service use*

	**GP visits**		**Specialist visits**		**Medication**^**1**^	
	**Under**	**Over**	**Under**	**Over**	**Under**	**Over**
	**n (%)**	**n (%)**	**n (%)**	**n (%)**	**n (%)**	**n (%)**
Age: mean years (sd)	66 (9.8)	62 (11.4)	65 (10.6)	64 (10.3)	71 (4.5)	68 (8.5)
Sex: Male	**27 (64)**	**4 (29)**	18 (60)	13 (38)	16 (67)	90 (47)
Female	**15 (36)**	**10 (71)**	12 (40)	21 (62)	8 (33)	102 (53)
Paid work: Yes	**4 (10)**	**7 (50)**	5 (17)	8 (23)	2 (8)	26 (14)
No	**38 (90)**	**7 (50)**	25 (83)	26 (76)	22 (92)	166 (86)
Income:						
≤ A$40,000	28 (67)	6 (42)	19 (64)	15 (44)	19 (68)	93 (75)
> A$40,000	10 (24)	6 (42)	8 (26)	14 (42)	3 (12)	39 (20)
Unknown	4 (9)	2 (1)	3 (10)	5 (15)	0 (0)	10 (5)
Living arrangement:						
Single	11 (26)	6 (43)	8 (27)	11 (32)	**14 (58)**	**64 (32)**
Couple	30 (71)	8 (57)	21 (70)	24 (65)	**10 (42)**	**122 (63)**
Other	1 (2)	0 (0)	1 (3)	1 (3)	**0 (0)**	**7 (4)**
Private health insurance:						
None	9 (21)	4 (29)	4 (13)	10 (29)	7 (29)	45 (23)
Any	28 (79)	10 (71)	26 (87)	24 (71)	17 (71)	147 (76)
Dukes Stage: A	6 (19)	3 (30)	7 (29)	6 (26)	0 (0)	19 (12)
B	12 (39)	4 (40)	8 (33)	9 (39)	8 (42)	58 (38)
C	13 (42)	3 (30)	9 (38)	8 (35)	11 (38)	76 (50)
Type of surgery						
None	**0 (0)**	**0 (0)**	**0 (0)**	1 (3)	0 (0)	0 (0)
Open	**25 (60)**	**8 (57)**	**20 (67)**	19 (56)	10 (42)	102 (53)
Laparoscopic	**17 (40)**	**6 (42)**	**10 (33)**	14 (41)	14 (58)	89 (46)
Polypectomy	**0 (0)**	**0 (0)**	**0 (0)**	0 (0)	0 (0)	1 (0.5)
Chemotherapy:						
No	23 (55)	7 (50)	19 (63)	16 (47)	**6 (25)**	**87 (45)**
Yes	19 (45)	7 (50)	11 (37)	18 (53)	**18 (75)**	**105 (55)**
Radiotherapy:						
No	34 (81)	13 (93)	28 (82)	24 (80)	21 (88)	162 (84)
Yes	8 (19)	1 (7)	6 (18)	6 (20)	3 (13)	30 (16)
Visit frequency:						
High^2^	**21 (50)**	**11 (79)**	**26 (76)**	**15 (50)**	-	-
Low	**21 (50)**	**3 (21)**	**8 (24)**	**15 (50)**	-	-
Intervention allocation:						
CanChange	23 (58)	7 (18)	16 (40)	20 (50)	19 (79)	111 (58)
Usual care	19 (53)	7 (19)	14 (39)	14 (39)	5 (21)	81 (42)

*Bolded figures indicate statistically significant at p < 0.05.

1. Medications were categorised into 4 groups: gastrointestinal, cardiovascular, psychological and COPD.

2. High frequency is defined by >3 GP visits or >4 specialist visits, and low frequency ≤3 GP visits and ≤4 specialist visits.

**Table 4:** Results of multivariate logistic regression (n = 76)

	**Odds Ratio**	**95% CI**	**P value**
**Model 1. GP agreement (no/yes)**			
Likelihood ratio chi^2^: 13.7, Adj R^2^: 0.16			
Age	1.04	0.97, 1.11	0.272
Employment category (working/not working)	4.35	1.04, 18.17	0.044
Gender (male/female)	0.29	0.09, 0.97	0.044
Frequency category (high/low)^1^	0.18	0.5, 0.63	0.007
**Model 2. Specialist agreement (no/yes)**			
Likelihood ratio chi^2^: 19.3, Adj R^2^: 0.29			
Age	1.00	0.91, 1.11	0.940
Surgery type (laparoscopic/open)	5.16	1.02, 26.11	0.047
Chemotherapy (no/yes)	0.67	0.10, 4.24	0.660
Frequency category (high/low)	0.10	0.01, 0.94	0.044

^1^ High GP visit frequency was > 3 visits.

^2^ High specialist visit frequency was defined as >4 visits.

### Specialist visits

**Figure 2:**
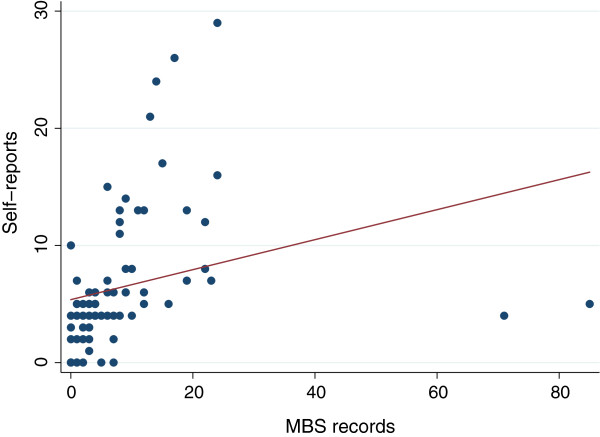
Scatterplot of frequency of self-reported specialist visits from telephone interview and MBS specialist visit records (n = 76).

The median number of visits for both MBS and self-reported specialist data was five however the mean difference was -2.18 showing a tendency for participants to under-report specialist visits by around two visits on average during the past six months. Perfect agreement was found in 12 (16%) participants, over-reporting in 34 (45%) participants and under-reporting in 30 (39%) participants (Table 2). When a margin of ±1 visit was allowed, 36% reported agreement with MBS records and 51% within ±2 visits (Table 2). The ICC was 0.73 (95%CI: 0.56, 0.91) indicating substantial agreement. The factors associated with over-reporting specialist visits were undergoing laparoscopic surgery and high visit frequency (>4 visits) in the previous six months (Table 3) however, only high frequency visits remained statistically significant in a logistic regression model (Table 4). Figure 2 shows greater clustering around fewer visits to specialists and two participants had extremely high numbers of records reported for specialist visits in the MBS data. When these outliers were excluded the spread of response pairs mirrors that of GP visits (not shown).

### Medication types

A total of 55/76 (72%) participants responded ‘yes’ to receiving any prescribed medications during the past six months. PBS records showed prescribed medications were provided to 67/76 (88%) of participants over the same time period. The kappa statistic for ‘any’ medication responses for the two data sources was 0.52 (SE 0.10, p < 0.001) or moderate agreement. Large discrepancies were found between self-reports and PBS data for these medication groups (Table 5). For the PBS data, 356 medicines were recorded for 76 participants compared to 323 recorded by self-reports, indicating medications were under-reported. However, within the four general medication groups of interest, under-reporting occurred in 4 (9%) of responses, over-reporting in 192 (76%) and 37 agreed absolutely (15%). The kappa statistic was 0.134 (SE 0.07, p = 0.029) indicating high disagreement. The only factors associated with more under-reporting included chemotherapy and living alone and no socio-demographic or treatment factors appeared to be linked with the extent of over-reporting medicines (Table 3).

**Table 5:** **Agreement between self-reported and administrative reports on selected medication categories (n = 253**^**1**^**)**

	**Survey n (%)**		**PBSn (%)**		**Difference n (%)**	
Gastrointestinal	69	35%	56	39%	13	23%
Cardiovascular	94	48%	60	42%	34	57%
Psychological	10	5%	18	13%	−8	−44%
COPD	22	11%	8	6%	14	175%
Total	195	100%	142	100%	53	37%

^1^ 253 responses from 76 participants were recorded for the 4 broad medication groups.

^2^ Kappa = 0.134, agreement 44% p = 0.029.

### Cost differences

Over six months, total costs estimated for GP visits for 76 participants were AU$13,962 for survey data versus AU$16,967 for MBS records. In bootstrapping analyses, the mean cost for GP visits for the survey data was AU$184 (95%CI: $142, $226) compared with AU$223 (95%CI: $182, $264) from MBS records and the mean difference was not statistically significant (p = 0.181). Corresponding total specialist costs were AU$27,253 for survey data versus AU$38,224 for MBS records. The bootstrapped mean costs for specialist visits were AU$359 (5%CI: $285, $433) for survey data and AU$503 (95%CI: $344, $662) for MBS records with no significant difference in means (p = 0.105).

## Discussion

In a sample of colorectal cancer survivors, the overall quality of self-reported doctors’ visits and medication use was mixed with reasonable agreement for doctors’ visits but poor agreement for medications. Although the ICCs and kappa statistics showed ‘substantial’ agreement between the two methods for doctors’ visits, considerable under-reporting occurred, the proportion of absolute agreement was small and the 95% confident limits ranged from poor to substantial. Nevertheless, when costs were applied to the survey data and compared with MBS fees, the cost differences were not substantially different. Medication types were considerably over-reported in our sample with the exception of psychological medicines that were under-reported.

Our results showed a tendency for males and those with frequent contacts to under-report actual visits, coinciding with the findings from other studies [3,5]. However, unlike other reports, we did not show disagreement was linked with older age, more severe disease and income or education levels for both doctors’ visits and medication use. Forty-three percent of our participants received laparoscopic surgery rather than radical open surgery and this was a factor associated with perfect agreement of specialist visits. Although the numbers involved are small, this may be a marker for participants having better general well-being and/or fewer side effects after surgery, and subsequently fewer services to recall, compared with participants undergoing other surgeries.

While our results point towards self-reported medication use being somewhat unreliable, it is important to understand the research context. Our participants have colorectal cancer and high treatment needs, they have multiple treatment modalities and they are likely to experience ongoing side-effects consistent with the evidence [12]. Treatment involves a protracted and sometimes complex navigation through the health system at different service locations [13]. Therefore, recall of health service utilisation over a six month period may be difficult for this group given their high usage and poor health status resulting in ‘poor agreement’ between self-reported and administrative data.

The agreement between the two methods for prescribed medication responses was disappointing despite efforts to ask participants to prepare records ahead of the interview time and clarify with the researcher any misunderstandings they had. However, this method assumed participants would be highly motivated which may have been unrealistic. Participants may have confused some prescription medications with over-the-counter medicines, participants may have included inexpensive medications not subsidised by the PBS or it is possible that hospital-acquired medications or those prescribed earlier in the recollection period were forgotten. It is clear from our findings that administrative data is necessary for collecting medication data in our participants receiving polypharmacotherapy. PBS data showed the total number of prescriptions (including repeats) was 1523 at a value of $244,231 to the government and $16,484 in out-of-pocket expenses for the participant. We did not test the reliability of frequency or repeats of specific prescriptions but it is clear from the survey responses for general groupings that this would have been too onerous for our participants to accurately recall.

Ultimately, the purpose of our study was to investigate whether self-reported health care utilisation via telephone surveys was an acceptable method for collecting data within a larger cost-utility analysis. If under-reporting service use can cancel out over-reporting and this occurs randomly across intervention arms, then minor discrepancies in resource use may be acceptable. However, our data suggested the extent of discrepancies was more serious however, there were no systematic differences in agreement categories across the CanChange intervention arms. When costs were applied to the frequency of doctor visits, MBS costs were significantly higher than costs from self-reported use, 18% higher for GP visits and 29% higher for specialist visits. Therefore, if the self-reported data is relied on for the cost-utility analysis, the extent of under-reporting may bias the final results. In this particular case, it may be preferable to use the mean cost and standard error from the Medicare records for use in modelling the larger study. Alternatively, the self-reported data collected on the full sample in the larger study could be increased by a compensating factor representing the extent of disagreement seen in our 76 participants.

Our study has several limitations. The small sample was not powered to detect significant differences in agreement category within logistic regressions. The framing of the survey questions may have been too broad and open to interpretation, particularly the medication question. To overcome this limitation we could have asked a closed question about medication usage including a list of specific drugs of interest (e.g., anti-depressants, anti-hypertensives) with their commonly available trade names. Finally, we assigned average costs of MBS attendance items to the survey data during our cost comparisons which may have obscured the variation in doctor services provided. However, the unit costs reflect the most common MBS items for GP and specialist visits and were considered reasonable estimates. Finally, important health service items collected by self-reports for allied health services, community services and home care were unable to be verified with Medicare records as they are not billable items and were therefore excluded from the analyses.

Research has suggested that cancer support services may promote downstream cost-savings to the health system from avoided GP visits and lower medication use [14]. The strength of this claim is not yet supported by the research evidence and the issue is further complicated by the measurement challenges for health service use. Due to having Medicare data on only a small sample of colorectal cancer survivors, we are not able to contribute meaningfully to this topic, however forthcoming research on a larger sample of men with prostate cancer receiving a psychological intervention [15] will inform this growing literature.

There is an increasing demand for data on health resource use at the patient level among researchers seeking reliable and high quality data. In comparative intervention research, the types and extent of resources used is important to evaluators to inform service delivery, future service planning and to contribute to assessments of cost-effectiveness.

## Conclusion

In conclusion, our findings suggest that self-reported data for doctors’ visits is reasonably reliable for use in economic studies but self-reported medication use is sub-optimal. Administrative data on doctor visits and medications are preferable to self-reported data due to substantial disagreement in self-reported telephone interviews. If self-reported data is the only available option, very specific closed questions for doctor visits and medication usage are recommended.

## Competing interests

None of the authors declare any competing interests.

## Authors’ contributions

LG and AH conceived of the study, and participated in its design and coordination. TP participated in the dataset management and analyses. LG performed the statistical analyses and drafted the manuscript. TP, AH and LG have contributed to, read and approved the final manuscript.

## Pre-publication history

The pre-publication history for this paper can be accessed here:

http://www.biomedcentral.com/1472-6963/12/440/prepub

## Supplementary Material

Additional file 1 CanChange Questionnaire - Time 1. Click here for file
