# Supplementary material for: Can colorectal cancer survivors recall their medications and doctor visits reliably?
Source: BMC Health Serv Res. 2012 Dec 2;12:440. doi: 10.1186/1472-6963-12-440 (PMC3536672; doi:10.1186/1472-6963-12-440)
Supplement: Additional file 1 — CanChange Questionnaire - Time 1. [file 1472-6963-12-440-S1.pdf]

Cancer Council Queensland

CanChange Questionnaire - Time 1

Instructions:

In addition to your telephone interview, we would like you to complete this short questionnaire to see how you are going. Please answer all of the questions. There are no “right” or “wrong” answers; we are only interested in what you think. All the information you provide will be kept strictly confidential.

Part A of this questionnaire includes questions on your feelings around your diagnosis of cancer, and the support you currently receive from family and friends. Part B includes questions about your medical history.

Please complete this Questionnaire before your telephone interview, so your telephone interviewer can help you if have any trouble answering the questions. After your telephone interview, it is important that you return this completed questionnaire to us in the reply paid envelope provided.

If you have any questions, please contact the lead investigator, Anna Hawkes on 3634 5305 or email annahawkes@cancerqld.org.au.

Thank you again for your participation in CanChange.

ID number	
Date returned	
Date entered	

PART A: This section includes questions on how you feel about your diagnosis of colorectal cancer, as well as the support you currently receive from friends and family.

Question 1. Please indicate how your cancer diagnosis has changed each of the statements below for you *(circle only one number for each statement)*

	**Not at all**	**Very small degree**	**Small degree**	**Moderate degree**	**Great degree**	**Very great degree**
1. My priorities about what is important in life	0	1	2	3	4	5
2. An appreciation for the value of my own life	0	1	2	3	4	5
3. I developed new interests	0	1	2	3	4	5
4. A feeling of self-reliance	0	1	2	3	4	5
5. A better understanding of spiritual matters	0	1	2	3	4	5
6. Knowing that I can count on people in times of trouble	0	1	2	3	4	5
7. I established a new path for my life	0	1	2	3	4	5
8. A sense of closeness with others	0	1	2	3	4	5
9. A willingness to express my emotions	0	1	2	3	4	5
10. Knowing I can handle difficulties	0	1	2	3	4	5
11. I'm able to do better things with my life	0	1	2	3	4	5
12. Being able to accept the ways things worked out	0	1	2	3	4	5
13. Appreciating every day	0	1	2	3	4	5
14. New opportunities are available which wouldn't have been otherwise	0	1	2	3	4	5
15. Having compassion for others	0	1	2	3	4	5
16. Putting effort into my relationships	0	1	2	3	4	5
17. I'm more likely to try to change things which need changing	0	1	2	3	4	5
18. I have a stronger religious faith	0	1	2	3	4	5
19. I discovered that I'm stronger than I thought I was	0	1	2	3	4	5
20. I learned a great deal about how wonderful people are	0	1	2	3	4	5
21. I accept needing others	0	1	2	3	4	5

Question 2. Please indicate how true each of these statements has been for you over the past 7 days *(circle only one number for each statement).*

**Additional Concerns**	**Not at all**	**A little bit**	**Some-what**	**Quite a bit**	**Very much**
1. I feel peaceful	0	1	2	3	4
2. I have a reason for living	0	1	2	3	4
3. My life has been productive	0	1	2	3	4
4. I have trouble feeling peace of mind	0	1	2	3	4
5. I feel a sense of purpose in my life	0	1	2	3	4
6. I am able to reach down deep into myself for comfort	0	1	2	3	4
7. I feel a sense of harmony within myself	0	1	2	3	4
8. My life lacks meaning and purpose	0	1	2	3	4
9. I find comfort in my faith or spiritual beliefs	0	1	2	3	4
10. I find strength in my faith or spiritual beliefs	0	1	2	3	4
11. My illness has strengthened my faith or spiritual beliefs	0	1	2	3	4
12. I know that whatever happens with my illness, things will be okay	0	1	2	3	4

Question 3. The following statements ask about the people in your life who provide you with help or support at the moment.

Each statement has two parts. For the first part, indicate how many people you know, *excluding yourself*, whom you can currently count on for help or support in the manner described. Put this answer in the darker column labelled "Number of People".

For the second part, circle how satisfied you are with the overall support you have received from these people (circle only one number for each statement). If there is no one who provides you with support for a statement, place "0" in the space provided, but still rate your level of satisfaction.

	**Number of People**	**Very satisfied**	**Fairly satisfied**	**A little satisfied**	**A little dissatisfied**	**Fairly dissatisfied**	**Very dissatisfied**
1. How many people can you really count on to be dependable when you need help?		6	5	4	3	2	1
2. How many people can you really count on to help you feel more relaxed when you are under pressure or tense?		6	5	4	3	2	1
3. How many people can you count on to accept you totally, including your worst and your best points?		6	5	4	3	2	1
4. How many people can you really count on to care about you, regardless of what is happening to you?		6	5	4	3	2	1
5. How many people can you really count on to help you feel better when you are feeling generally down-in-the-dumps?		6	5	4	3	2	1
6. How many people can you count on to console you when you are very upset?		6	5	4	3	2	1

PART B: This section includes questions on your medications and your recent visits to doctors and other health professionals.

Your telephone interviewer will run through these questions with you if you have any trouble answering them, however please try to collect all of this information and answer these questions before your telephone interview.

Question 4. Are you currently taking any medications that have been prescribed for you by a doctor or specialist?

☐ Yes
☐ No

If yes, please provide details *(name of the medication and the dose per day you are taking)*

Medication Name	Total dose per day
Example: Maxolon	*Example: 10mg three times/day when necessary*

Question 5. Have you visited a doctor (general practitioner or specialist) in the last 6 months?

☐ Yes

☐ No (go to question 6)

Please provide details *(doctor name, doctor type, suburb of the doctor's practice and the number of times you have visited that particular doctor)*

Doctor Name	Doctor Type	Practice Name	Suburb	Number of Times Visited
Eg: Dr. Smith	*Oncologist*	*Johns Medical Centre*	*Chermside*	*12*

Question 6. Have you had an appointment with any other health professional (eg. dietitian, physiotherapist, psychologist, naturopath etc.) over the past 6 months? This does not include hospital outpatient services.

☐ Yes

☐ No (Go to question 7)

Please provide details *(type of health professional, reason for the visit and the number of times you have visited that particular health professional)*

Type of Health professional	Reason	Number of times visited
Eg. Stoma nurse	*Stoma management*	*2*

Question 7. Have you been admitted to hospital since your diagnosis of colorectal cancer?

☐ Yes
☐ No (Go to question 8)

Please provide details*.(hospital name, reason for visiting the hospital, the dates of admission or number of admissions, as well as your date of discharge)*

Hospital Name	Reason for the hospital visit	Date of admission(s) (or number of admissions) to hospital	Date of discharge from hospital
Eg: Wesley	*Nausea and vomiting*	*02/03/09*	*06/3/09*

Question 8. Have you used any other <u>hospital service</u> over the past 6 months that did not involve an admission? (for example: emergency clinic, outpatient services, day procedures including chemotherapy treatment etc.)

☐ Yes
☐ No (Go to question 9)

Please provide details *(type of hospital or clinic service you attended, the name of the of the hospital or clinic, the reason for the visit and the number of time you accessed this service)*

Type of Hospital or clinic service	Hospital or clinic name	Reason for the hospital or clinic visit	Number of times accessed
Eg: Chemotherapy	*Wesley*	*Chemotherapy treatment*	*6 sessions*

Question 9. Are there any other health services you have used over the past 6 months? (For example: blue care, meals on wheels, home visits by health care workers / nurses)?

☐ Yes
☐ No

If yes please provide details *(type of service you used, the reason for visiting the service and number of times you used this service).*

Type of Health Service	Name of service	Reason for service	Number of times accessed
Eg: Home visits by health care workers	*Blue Care*	*Feeling unwell*	*2*

Thank you for completing this questionnaire. Please remember to return it in the reply paid envelope.
